# The stepped wedge trial design: a systematic review

**DOI:** 10.1186/1471-2288-6-54

**Published:** 2006-11-08

**Authors:** Celia A Brown, Richard J Lilford

**Affiliations:** 1 Department of Public Health and Epidemiology, The University of Birmingham, Birmingham, UK

## Abstract

**Background:**

Stepped wedge randomised trial designs involve sequential roll-out of an intervention to participants (individuals or clusters) over a number of time periods. By the end of the study, all participants will have received the intervention, although the order in which participants receive the intervention is determined at random. The design is particularly relevant where it is predicted that the intervention will do more good than harm (making a parallel design, in which certain participants do not receive the intervention unethical) and/or where, for logistical, practical or financial reasons, it is impossible to deliver the intervention simultaneously to all participants. Stepped wedge designs offer a number of opportunities for data analysis, particularly for modelling the effect of time on the effectiveness of an intervention. This paper presents a review of 12 studies (or protocols) that use (or plan to use) a stepped wedge design. One aim of the review is to highlight the potential for the stepped wedge design, given its infrequent use to date.

**Methods:**

Comprehensive literature review of studies or protocols using a stepped wedge design. Data were extracted from the studies in three categories for subsequent consideration: study information (epidemiology, intervention, number of participants), reasons for using a stepped wedge design and methods of data analysis.

**Results:**

The 12 studies included in this review describe evaluations of a wide range of interventions, across different diseases in different settings. However the stepped wedge design appears to have found a niche for evaluating interventions in developing countries, specifically those concerned with HIV. There were few consistent motivations for employing a stepped wedge design or methods of data analysis across studies. The methodological descriptions of stepped wedge studies, including methods of randomisation, sample size calculations and methods of analysis, are not always complete.

**Conclusion:**

While the stepped wedge design offers a number of opportunities for use in future evaluations, a more consistent approach to reporting and data analysis is required.

## Background

Randomised Controlled Trials (RCTs) are considered the 'Gold Standard' test of clinical effectiveness [1] and such trials are increasingly being used in evaluations of non-clinical interventions. There are many ways of classifying RCTs, such as the extent of blinding, method of randomisation (including whether interventions will be randomised at individual or cluster level) and the inclusion (or not) of a preference arm [2]. A further classification is the way in which participants are exposed to the intervention [2] and we refer to this as the 'design' of the RCT. This paper provides a review of studies employing a particular design, known as a 'stepped wedge'.

**Figure 1:**
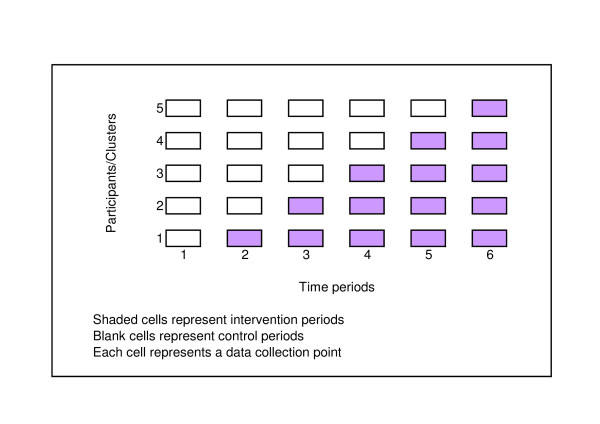
Example of a stepped wedge study design.

In a stepped wedge design, an intervention is rolled-out sequentially to the trial participants (either as individuals or clusters of individuals) over a number of time periods. The order in which the different individuals or clusters receive the intervention is determined at random and, by the end of the random allocation, all individuals or groups will have received the intervention. Stepped wedge designs incorporate data collection at each point where a new group (step) receives the intervention. An example of the logistics of a stepped wedge trial design is shown in Figure 1, which shows a stepped wedge design with five steps. Data analysis to determine the overall effectiveness of the intervention subsequently involves comparison of the data points in the control section of the wedge with those in the intervention section.

Cook and Campbell were possibly the first authors to consider the potential for *experimentally staged introduction *in a situation when an innovation cannot be delivered concurrently to all units [3]. The first empirical example of this design being employed is in the Gambia Hepatitis Study, which was a long-term effectiveness study of Hepatitis B vaccination in the prevention of liver cancer and chronic liver disease [4]. It is from this latter study that we have taken the term 'stepped wedge'.

There are two key (non-exclusive) situations in which a stepped wedge design is considered advantageous when compared to a traditional parallel design. First, if there is a prior belief that the intervention will do more good than harm [5], rather than a prior belief of equipoise [6], it may be unethical to withhold the intervention from a proportion of the participants, or to withdraw the intervention as would occur in a cross-over design. Second, there may be logistical, practical or financial constraints that mean the intervention can only be implemented in stages [3]. In such circumstances, determining the order in which participants receive the intervention at random is likely to be both morally and politically acceptable and may also be beneficial for trial recruitment [7]. An example of an intervention where a stepped wedge design may be appropriate in evaluation is a school-based anti-smoking campaign that is delivered by one team of facilitators who travel to each participating school in turn.

It is also important to note that the stepped wedge design is likely to lead to a longer trial duration than a traditional parallel design, particularly where effectiveness is measured immediately after implementation. The design also imposes some practical implementation challenges, such as preventing contamination between intervention participants and those waiting for the intervention and ensuring that those assessing outcomes are blind to the participant's status as intervention or control to help guard against information bias. Blinding assessors is particularly important since it is almost impossible to blind participants or those delivering the intervention, since both will be aware of the 'step' from control to intervention status. As will be shown in this paper, a variety of approaches to statistical analysis have been employed empirically, in part due to the complex nature of the analysis itself.

The potential benefits of employing a stepped wedge design can be illustrated by considering the £20 m evaluation of the Sure Start programme in the UK [8]. The Department for Education and Skills ruled out a cluster trial where the deprived areas identified as in need of Sure Start would be randomised to either receive the intervention or act as controls, since to intervene in some areas but not in others was judged unacceptable. The evaluation has instead used a non-randomised control group, consisting of 50 "Sure Start-to-be" communities, compared to the 260 Sure Start intervention communities. However the local programmes, evaluated by Belskey et al. [9] were in fact introduced in six waves between 1999 and 2003 [10]. This implementation strategy actually provided an excellent opportunity for a stepped wedge study design, which would have met both ethical and scientific imperatives. Determining the order in which communities received the intervention at random would have been demonstrably impartial and hence a fair way to allocate resources. From the scientific point of view, randomisation would eliminate allocation bias and the stepped wedge deign would have offered a further opportunity to measure possible effects of time of intervention on the effectiveness of the intervention. Since no pre-intervention measurements were undertaken, it was also impossible to separate the effects of the Sure Start programmes from any underlying temporal changes within each community and these effects could also have been investigated through the use of a stepped wedge design.

In this paper, we provide a review of studies employing a stepped wedge design. The review includes studies based on both individual and cluster allocations and is not restricted to RCTs. Indeed, we do not apply any methodological filters. The aim of the review is to determine the extent to which the stepped wedge design has been employed empirically and, for the available studies, to examine the background epidemiology, why researchers decided on a stepped wedge design and methods of data analysis. The review is intended to be systematic and includes trials from all fields. However we would be interested to hear about any further examples that our search may have missed.

## Methods

### Search strategy

We searched the Current Controlled Trials Register, the Cochrane Database, Medline, Cinahl, Embase, PsycInfo, Web of Knowledge and Google Scholar for papers and trial protocols using the following phrases: step wedge, stepped wedge, experimentally staged introduction and the nine possible combinations of incremental/phased/staggered and recruitment/introduction/implementation. The search was undertaken in October 2005 and repeated in March 2006. In addition, we checked the citations of the original Gambia Hepatitis Study and the references and citations of other relevant papers. We included any relevant papers in the English language that employed a stepped wedge design (although as indicated by the search terms the authors may have used an alternative term to describe their trial design), but did not include any date or subject restrictions. We exclude multiple baseline designs, which are generally applied to analyse the response of single subjects to an intervention and where analysis is undertaken separately for each individual, since exposure to the intervention is often delayed until a stable baseline has been achieved rather than being determined randomly [11]. Given our focus on study design, we exclude any follow-up papers presenting further results and analysis of a study already included in the review.

### Data extraction and analysis

Data were extracted from studies included in the review onto a standard proforma (see Additional file 1) in three main categories: basic information about the trial (including epidemiology, intervention and trial size), reasons for using a stepped wedge design and methods of data analysis. Data were transposed from the proformas to a database that was subsequently interrogated to elicit summary information and key themes in each of the three categories across all of the studies.

## Results and discussion

### Yield

**Figure 2:**
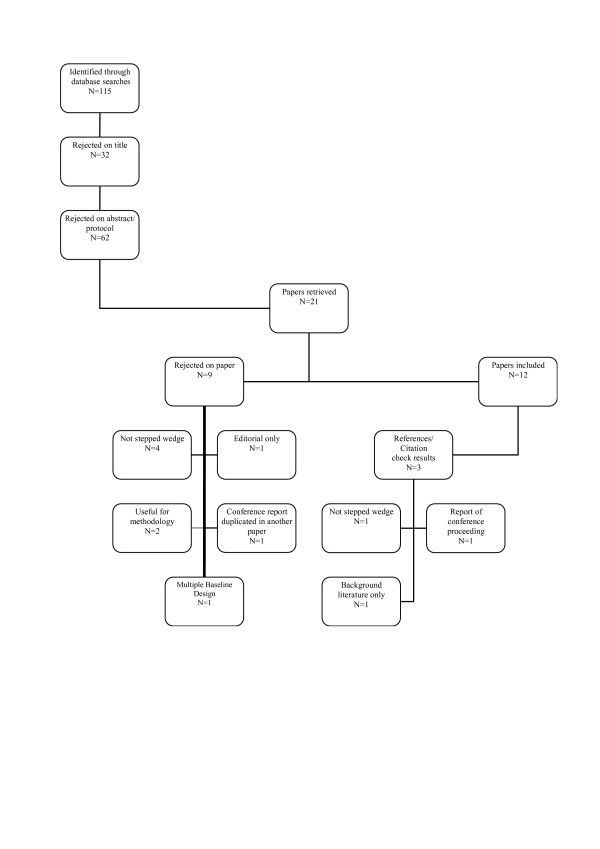
Results of literature search.

Figure 2 shows the results of our search which identified only 12 papers or protocols (referred to as studies) in which a stepped wedge design was described. The studies included in the review described their study design as either stepped wedge or phased introduction/implementation. We contacted the authors of the conference proceeding report identified by a citation check and a protocol identified by the Controlled Trials Register but were unable to obtain sufficient information about these trials to include them in our review. Three of the included studies [12-14] are protocols describing trials that were being designed or implemented rather than providing results of the evaluation. Basic information about each of the included studies is shown in Tables 1, 2, 3.

**Table 1:** Study epidemiology

Lead Author	Date	Disease	Country	Setting
Gambia Hepatitis Study Group [4]	1987	Liver cancer	Gambia	Regions
Cook [17]	1996	Substance abuse	USA	Workplace
Wilmink [19]	1999	Ruptured abdominal aortic aneurysms	UK	GP surgeries
Somerville [14]	2002	Respiratory Health	UK	Houses in Watcombe
Fairley [15]	2003	HIV (Adherence to antiretroviral therapy)	Australia	Sexual health clinic
Hughes [12]	2003	HIV (Mother to child transmission)	Zambia and Uganda	Health clinics
Levy [16]	2004	HIV (Adherence to antiretroviral therapy)	Australia	Ambulatory care clinic in a tertiary hospital
Priestly [22]	2004	Critical care	UK	NHS hospital trust
Bailey [18]	2004	Water-borne diseases	South Africa	Households
Grant [20]	2005	TB in HIV+ men	South Africa	Company health centre
Ciliberto [21]	2005	Childhood malnutrition	Malawi	National rehabilitation units
Chaisson [13]	2005	TB in HIV+ men	Brazil	HIV clinics

**Table 2:** Study interventions

Lead Author	Date	Nature of Intervention	Level of 'stepping'	Is 'stepping' randomised?	Primary outcome measure
Gambia Hepatitis Study Group [4]	1987	Hepatitis B vaccination	Vaccination team	Yes	Liver cancer rates/Vaccine efficacy
Cook [17]	1996	Educational programme	Cohort	Yes	Health Behaviour Questionnaire measures
Wilmink [19]	1999	Screening programme	Surgery and Individual	Surgery – not stated; individual – yes	Incidence and mortality of RAAAs
Somerville [14]	2002	Improvements to housing	Sets of houses	Yes	Respiratory Health Symptoms
Fairley [15]	2003	Education programme and individual plans	Not stated	Yes	Proportion of missed doses
Hughes [12]	2003	Provision of Nevirapine to pregnant HIV+ women	Pre-natal clinic	Not stated	Mother to child HIV transmission
Levy [16]	2004	Education programme and individual plans	Individual	Yes	Proportion of missed doses
Priestly [22]	2004	Critical care outreach teams	Ward	Yes – in pairs	Rate of in-hospital deaths
Bailey [18]	2004	Provision of piped water to house yards	District	Not stated	Water quality
Grant [20]	2005	Screening and Isoniazid therapy	Individual	Yes	TB episodes >90 days after clinic entry
Ciliberto [21]	2005	Home-based therapy with ready to use therapeutic food	Rehab unit	Not stated	Attainment WHZ score >-2/Death
Chaisson [13]	2005	Screening and treatment for TB	Clinic	Yes	TB Incidence

**Table 3:** Study size

Lead Author	Date	Number of 'steps'	Number of participants:		Time period between steps
			**Intervention**	**Control**	

Gambia Hepatitis Study Group [4]	1987	17	61,065	63,512	10–12 weeks
Cook [17]	1996	2	371		Not stated
Wilmink [19]	1999	Individual – 13,147	29,713 person years	70,298 person years	Not stated – total 6 years
Somerville [14]	2002	2	119		1 year
Fairley [15]	2003	43	43		Not stated – total 20 weeks
Hughes [12]	2003	2	Aim: 304	Aim: 304	7 months (4 intervention, 3 wash-out)
Levy [16]	2004	Not stated (2 randomisation periods)	68		Not stated – total 20 weeks
Priestly [22]	2004	8	2,903	4,547	4 weeks
Bailey [18]	2004	4	400		About 3 months
Grant [20]	2005	1,655	1,655		Not stated – total 26 months
Ciliberto [21]	2005	7	992	186	3 weeks
Chaisson [13]	2005	29	Not stated		1 month

Notes:

The number of individuals is based on recruitment to the trial, rather than completed follow-up numbers.

Where only one figure for the number of participants is given, each individual/household participant receives the intervention at some stage during the trial.

In the Wilmink study [19], individuals cross-over from control to intervention at various points, but contribute person-years of data to both sections of the wedge.

In the remaining four studies with both 'intervention' and 'control' participants [4, 12, 21, 22], the unit of randomisation is the clinic or ward and hence an individual visiting the clinic/ward while it is in the control section of the wedge will not receive the intervention. Individuals visiting the clinic/ward once it has crossed-over to intervention will then contribute data to the intervention section of the wedge.

### Epidemiology and study details

While the small sample makes generalisations difficult, the stepped wedge design appears to be primarily used in evaluating interventions in developing countries, with HIV the most common disease addressed (Table 1). Table 2 identifies that a number of different interventions were being evaluated, with vaccination, screening and education plans emerging as the most common interventions. Such interventions are likely to have an existing evidence base, adding to intuitive beliefs that the intervention is likely to do more good than harm. It is also possible that the use of the stepped wedge design is increasing, with 9 (75%) of the studies published since 2002.

As shown in Table 2, three of the studies were randomised (and thus stepped) at the level of the individual and we suspect that Fairley et al. [15] also applied individual-level randomisation, since this study is a precursor to another similar study [16]. The remaining eight studies are cluster trials, with houses, clinics, wards and districts receiving the intervention in each time period. Of the cluster studies, three [14,17,18] are cohort designs (with the same individuals in each cluster in the pre and post intervention steps) and the remainder are repeated cross-section designs (with different individuals in each cluster in the pre and post intervention steps). It is only permissible to include terminal end-points (such as death) in studies with a repeated cross-sectional design: in individual/cohort designs if a participant has died in the control phase, it is impossible for them to die in the intervention stage. Nevertheless, this axiom is violated in one of the cohort designs [19] where incidence and mortality from ruptured abdominal aortic aneurysms are used as end-points. This problem is likely to introduce a "healthy survivor" bias [20].

Two of the cluster studies [12,18] did not indicate whether stepping was randomised. In addition, it is not clear whether the order in which Nutritional Rehabilitation Units were allocated to the intervention was determined at random in the study reported by Ciliberto et al. [21], although this is unlikely given that the paper suggests that randomised assignment in which some clusters would not receive the intervention was not possible due to resource constraints and cultural beliefs. However, of the remaining nine studies, only three [14,20,22] provided any detail on the method of randomisation employed and none of these would have fulfilled all of the requirements relating to randomisation (sequence generation, allocation concealment and implementation) detailed in the CONSORT statement or its extension for cluster studies [23,24].

As noted above, it is almost impossible to blind participants or those involved in delivering the intervention from being aware of whether a participant is currently in the control or intervention section of the wedge. This makes blinding of those assessing outcomes particularly important in protecting against information biases, particularly where outcomes are subjective. None of the studies in the sample provided enough detail to determine whether outcome assessments were blinded, with one study [14] deciding not to blind assessors to help maintain response rates. The intervention in this study involved improvements to housing, with health and environmental assessments undertaken in participants' homes so that participants would not have to travel to a 'neutral' location.

The studies varied considerably in terms of number of steps and number of participants (Table 3). It may be relevant to question whether there is a minimum number of steps required for the trial to be classed as a stepped wedge. Three of the studies [12,14,17] include only two steps. In two of these studies [14,17] participants are randomised to two cohorts, with one cohort receiving the intervention while the other cohort served as the control. The control groups subsequently receive the intervention and further evaluation of effectiveness was undertaken, suggestive of the stepped wedge approach. The third study [12] employs what the authors term a "combined parallel/stepped wedge design", although only four out of the eight clusters crossed-over from the control to the intervention groups, with all of these clusters crossing-over at the same time. The mean number of steps in the remaining five cluster studies was 13 (range 4–29). Sample size calculations are reported in just five studies [4,12,13,21,22].

### Motivations for employing a stepped wedge design

All of the studies bar one [15] identified one or more motivations for employing a stepped wedge design, although the level of detail regarding motivations varied. Four studies [16,17,20,21] reported using a stepped wedge design to prevent ethical objections arising from withholding an intervention anticipated to be beneficial. Practical difficulties with providing the intervention to everyone simultaneously were mentioned in four studies, due to insufficient resources in three [4,14,21] and logistical difficulties in two [4,18]. The authors reported a desire to use an RCT for evaluation in four studies [4,14,17,22] and scientific reasons were given in five studies: allowing individuals/clusters to act as their own controls [12,19] and to detect underlying trends/control for time [12,13,20,21].

### Methods of data analysis

No two studies use the same methods of analysing data, although most compare outcomes in the intervention and control sections of the stepped wedge across the entire data set. The primary method(s) of data analysis for each study are shown in Table 4. These methods vary considerably in terms of their complexity and there is insufficient information to determine the appropriateness of each method. Only two studies [13,14] propose a cost-effectiveness analysis. The two studies that apply step-by-step analysis [4,13] provide a separate analysis for each step in the trial, in order to separate out underlying time trends. Of the remaining studies that reported using a stepped wedge design to control for underlying time trends, Grant et al. [20] apply a Poisson random effects model to control for disease progression and Ciliberto et al. [21] use linear and logistic models to consider the effect of month (as a seasonal effect), although the results are not reported. The Hughes et al. [12] protocol includes time as a component in the model, but the analytical approach to be taken is not clear. Cook et al. [17] and Priestly et al. [22] also consider the impact of time on effectiveness. The latter uses matched pairs of wards to control for inter-temporal changes, randomising one ward in each pair to early intervention and one to late intervention. Analysis is then undertaken using a sub-set of three 4-week time periods for each ward pair, comparing the outcomes in intervention and control wards across the same 12-week period.

**Table 4:** Methods of analysis

Lead Author	Date	Primary outcome measure	Method(s) of Analysis
Gambia Hepatitis Study Group [4]	1987	Liver cancer rates/Vaccine efficacy	Comparisons of incidence rates on a step by step basis to identify vaccine efficacy
Cook [17]	1996	Health Behaviour Questionnaire measures	Comparison of group means and group by time, gender and education interactions (F-test)
Wilmink [19]	1999	Incidence and mortality of RAAAs	Poisson likelihood distribution for incidence rates in person years and maximum likelihood rate ratios
Somerville [14]	2002	Respiratory Health Symptoms	Not stated (description of intervention only)
Fairley [15]	2003	Proportion of missed doses	Unpaired t-test of means
Hughes [12]	2003	Mother to child HIV transmission	Not stated (protocol only)
Levy [16]	2004	Proportion of missed doses	Wilcoxon rank-sum test
Priestly [22]	2004	Rate of in-hospital deaths	Logistic regression Cox proportional hazard models (length of stay)
Bailey [18]	2004	Water quality	Summary statistics only Time series analysis for diarrhoea rates
Grant [20]	2005	TB episodes >90 days after clinic entry	Poisson random effects model
Ciliberto [21]	2005	Attainment WHZ score >-2/Death	95% CI for differences between groups Linear and logistic regression for effects of covariates
Chaisson [13]	2005	TB Incidence	Step by step analysis of incidence Conditional logistic regression Cost-effectiveness analysis

Four of the studies [4,12,19,21] included long term follow-up beyond the period in which stepping took place, since the outcomes to be assessed may occur at a point following the final step (i.e. there is a lag between intervention and outcome).

## Conclusion

Our review has identified a number of evaluations where a stepped wedge design was clearly appropriate and where the design can provide sound evidence to guide future practice. However not all of the studies included here would fulfil the methodological requirements for a controlled trial and hence we propose that if a stepped wedge design is to be applied, authors should register their trial on the Controlled Clinical Trials Register and follow appropriate reporting guidelines, such as the CONSORT statement or its equivalent for cluster trials [23,24]. A particular concern is with the lack of blinding of those assessing subjective outcomes. This is important given the difficulties associated with blinding participants and those delivering the intervention from their status as intervention or control, since it will nearly always be evident to both groups when the step from control to intervention occurs.

The heterogeneity of analytical methods applied in the studies suggests that a formal model considering the effects of time would help others planning a stepped wedge trial and we will present our exposition of such a model in a subsequent paper. A recent paper by Hussey and Hughes [25] also provides detail regarding the analysis of stepped wedge designs. Two particular statistical challenges are controlling for inter-temporal changes in outcome variables and accounting for repeated measures on the same individuals over the duration of the trial.

The opportunities arising from modelling the effects of time can be illustrated by considering the stepped wedge design as a multiple arm parallel design, in which the research aims not only to assess intervention effects, but also to determine whether time of intervention (at the extremes intervening early as opposed to intervening late) impacts the effectiveness of the intervention. Such time effects can also include seasonal variations and disease progression: the latter may be particularly relevant when evaluating interventions targeting treatments for HIV. Although a traditional parallel trial design can be used to examine general secular trends it cannot explore the particular relationship between time of intervention and effectiveness.

One limitation of our review is the possibility that our search strategy did not identify all of the studies that have employed a stepped wedge design. In particular, we may have missed 'delayed intervention' studies with two steps, in which the delayed group receive the intervention after the outcomes from initial intervention group have been evaluated, but where the outcomes of the delayed group are also evaluated. We have included three studies of this nature in this review [12,14,17] although it is questionable whether studies with only two steps should be considered as stepped wedge designs. One reason for this is the limitations for generalisation, particularly with respect to the impact of time on effectiveness.

Considering the scientific advantages of the stepped wedge design, it has rarely been used in practice and hence we advocate the design for evaluating a wide range of interventions, although we are not the first to do so [26-28]. In terms of interventions likely to do more good than harm, a stepped wedge design may be particularly beneficial in evaluating interventions being implemented in a new setting, where evidence for their effectiveness in the original setting is available, or for patient safety interventions that have undergone careful pre-implementation evaluation to rule out any collateral damage. The stepped wedge design may also be appropriate for cost-effectiveness analyses of interventions that have already been shown to be effective. However, the stepped wedge design requires a longer trial duration than parallel designs and also presents a number of challenges, including both practical and statistical complexity. Hence careful planning and monitoring are required in order to ensure that a robust evaluation is undertaken.

## List of Abbreviations

RCT Randomised Controlled Trial

## Competing interests

The author(s) declare that they have no competing interests.

## Authors' contributions

CB and RL designed the study, CB undertook the literature searching, data extraction and analysis and CB and RL drafted the manuscript. Both authors read and approved the final manuscript.

## Pre-publication history

The pre-publication history for this paper can be accessed here:



## Supplementary Material

Additional file 1 Data extraction proforma. Proforma used to extract data from the included papers or protocols prior to generating a database from these data. Click here for file
