# Supplementary material for: The stepped wedge trial design: a systematic review
Source: BMC Med Res Methodol. 2006 Nov 8;6:54. doi: 10.1186/1471-2288-6-54 (PMC1636652; doi:10.1186/1471-2288-6-54)
Supplement: Additional file 1 — Data extraction proforma. Proforma used to extract data from the included papers or protocols prior to generating a database from these data. [file 1471-2288-6-54-S1.doc]

**Additional Data File 1: Data Extraction Proforma**
